# Efficacy of stimulus discrimination training for reducing unwanted memories in student journalists

**DOI:** 10.1080/20008066.2025.2558385

**Published:** 2025-10-03

**Authors:** Gabriella Tyson, Anke Ehlers, Jennifer Wild

**Affiliations:** a Department of Experimental Psychology, University of Oxford, Oxford, UK; b Oxford Health NHS Foundation Trust, Oxford, UK; c Department of Psychiatry, Phoenix Australia Centre for Posttraumatic Mental Health, University of Melbourne, Melbourne, Australia

**Keywords:** PTSD, prevention, intrusive memories, stimulus discrimination, memory suppression, journalists, digital intervention, TEPT, recuerdos intrusivos, discriminación de estímulos, supresión de recuerdos, intervención digital, periodistas, prevención

## Abstract

**Background:** Journalists are frequently exposed to traumatic images and events, which may contribute to poor mental health, especially in those starting in the profession. Evidence-based preventative tools are needed to reduce the effects of exposure to these occupational stressors. Previous research demonstrates that the strategy journalists most commonly apply to traumatic images is suppression.

**Objective:** This experiment investigated whether stimulus discrimination, a technique used in cognitive therapy for PTSD (CT-PTSD; Ehlers et al., 2005. Cognitive therapy for post-traumatic stress disorder: development and evaluation. *Behaviour Research and Therapy*, *43*(4), 413–431) for reducing intrusive trauma memories, is more effective than memory suppression.

**Methods:** Student journalists were randomly allocated to one session of online training in stimulus discrimination (*N* = 34; Mage = 23.65, SD = 4.18; 24 female) or suppression (*N* = 34; Mage = 24.26, SD = 4.55; 24 female) before exposure to analogue trauma film clips. Participants then completed daily diaries of intrusive memories of the film clips for one week and completed PTSD symptom measures at one-week follow-up.

**Results:** Compared to participants trained in memory suppression, those trained in stimulus discrimination reported significantly fewer intrusive memories, less distress associated with intrusions and lower PTSD symptom severity at follow-up. There were no training-specific effects associated with depression or resilience at follow-up.

**Conclusions:** The study found that student journalists can be trained in stimulus discrimination and that this CT-PTSD tool significantly reduced intrusive memories and associated PTSD symptoms after post-training exposure to traumatic images.

## HIGHLIGHTS

- Working as a journalist can involve exposure to traumatic events and increase risk for mental ill health.
- Journalists most often respond to intrusions of stressful experiences using strategies, such as memory suppression, that are understandable but may be unhelpful.
- Training journalists in stimulus discrimination prior to exposure to analogue trauma led to significantly fewer intrusive memories, less associated distress and fewer self-reported PTSD symptoms at 1 week follow-up compared to journalists trained in memory suppression.
- Stimulus discrimination effectively reduced intrusive memories and associated symptoms.
- Stimulus discrimination may offer protective benefits against developing PTSD and may have promise as a prevention tool.

## Abstract (es)

**Antecedentes:** Los periodistas están con frecuencia expuestos a imágenes y eventos traumáticos, lo que puede contribuir a un deterioro de la salud mental, especialmente en quienes recién comienzan en la profesión. Se requieren herramientas preventivas basadas en evidencia para reducir los efectos de la exposición a estos estresores laborales. Investigaciones previas demuestran que la estrategia más comúnmente aplicada por los periodistas frente a imágenes traumáticas es la supresión.

**Objetivo:** Este experimento investigó si la discriminación de estímulos, una técnica utilizada en la terapia cognitiva para el TEPT (TC-TEPT; Ehlers et al., 2005) para reducir recuerdos intrusivos de trauma, es más eficaz que la supresión de recuerdos.

**Método:** Estudiantes de periodismo fueron asignados aleatoriamente a una sesión de entrenamiento en línea de discriminación de estímulos (*N* = 34; Medad = 23,65, DE = 4,18; 24 mujeres) o de supresión (*N* = 34; Medad = 24,26, DE = 4,55; 24 mujeres) antes de la exposición a fragmentos de películas con trauma análogo. Posteriormente, los participantes completaron registros diarios de recuerdos intrusivos de los fragmentos durante una semana y respondieron medidas de síntomas de TEPT en un seguimiento a una semana.

**Resultados:** En comparación con los participantes entrenados en supresión de recuerdos, aquellos entrenados en discriminación de estímulos reportaron significativamente menos recuerdos intrusivos, menor malestar asociado a las intrusiones y una menor severidad de síntomas de TEPT en el seguimiento. No se observaron efectos específicos del entrenamiento en relación con depresión o resiliencia en el seguimiento.

**Conclusiones:** El estudio encontró que los estudiantes de periodismo pueden ser entrenados en discriminación de estímulos y que esta herramienta de la TC-TEPT redujo significativamente los recuerdos intrusivos y los síntomas asociados al TEPT tras la exposición post-entrenamiento a imágenes traumáticas.

## 1. Introduction

Journalists report high levels of exposure to trauma (MacDonald et al., 2017), resulting in high rates of posttraumatic stress disorder symptoms (PTSD) (Feinstein & Nicolson, 2005) and poorer wellbeing. However, there is little research into preventative interventions to reduce mental ill-health in the occupation, leaving the industry turning to less effective, non-evidence-based support (Šimunjak & Menke, 2023). The hallmark feature of PTSD is intrusive memories of the trauma (American Psychiatric Association, 2013), i.e. sensory impressions and emotional responses (e.g. horror, fear, sadness or shame) from the traumatic event, that are retrieved without much context or time perspective (Ehlers et al., 2004; Michael et al., 2005). Distressing intrusive memories can motivate the individual to avoid reminders of the trauma and are often linked to negative thoughts about the self or the world and symptoms of hyperarousal, such as feeling on edge, easily startled, with disrupted sleep and poor concentration. Preventing or reducing intrusive memories may prevent the development of PTSD (Iyadurai et al., 2018).

Given the high rates of exposure to trauma it is unsurprising that journalists experience frequent intrusive memories of traumatic events that they witness or report on in their line of work (Weidmann & Papsdorf, 2010). Research suggests that journalists typically respond with suppression, a strategy that aims to push the memory out of mind (Tyson & Wild, 2021). Empirical studies suggests that suppression of unwanted memories can paradoxically increase their frequency (known as the rebound effect) (Abramowitz et al., 2001) and this effect has also been demonstrated for trauma memories in PTSD (Shipherd & Beck, 2005). It is also a difficult strategy to maintain over long periods (Van Schie & Anderson, 2017) and thus may be an inefficient way of coping with unwanted memories of traumatic events. In contrast, a limited number of studies found that suppression may help keep negative thoughts at bay (Anderson & Green, 2001; Hu et al., 2017). In one study (Mamat & Anderson, 2023) participants trained in suppression of fearful thoughts reported less vivid fearful thoughts and images than a control group trained to suppress neutral thoughts, and also showed lower anxiety and negative affect three months later. Other studies have utilised think/no-think paradigms, which involve suppression of words during retrieval attempts, where participants are shown cues and instructed to think of the associated word or to avoid thinking of it (Anderson & Green, 2001; Hu et al., 2017). In these paradigms, suppression of target words led to diminished recall, leading researchers to conclude that suppression reduces memory accessibility. This suggests that suppression can inhibit recall of previously suppressed items, which could indicate that in some contexts, suppression leads to retrieval-induced forgetting rather than producing a rebound effect. It is, however, unclear to what extent these findings apply to memories of traumatic material. Many stimuli in everyday life can trigger distressing intrusive trauma memories. These triggers usually have some resemblance to sensory impressions experienced or witnessed during the trauma, e.g. a particular colour, sound or smell (American Psychiatric Association, 2013). Ehlers and Clark (Ehlers & Clark, 2000) proposed that two basic learning mechanisms contribute to the easy triggering of intrusive memories by such perceptually similar cues: (1) strong perceptual priming and (2) associative learning in which stimuli become associated with strong affective and physiological responses, which can generalise to other similar stimuli. There is experimental evidence for both learning mechanisms (e.g. Kaczkurkin et al., 2017; Sündermann et al., 2013). Both have the effect that after trauma, stimuli that are different from those during the trauma, but share sensory similarity, can trigger unwanted memories of the traumatic event and associated emotional reactions.

These findings led to the development of a stimulus discrimination training technique used in cognitive therapy for PTSD to reduce involuntary triggering of intrusive trauma memories in everyday life (Ehlers et al., 2005). Patients first learn to identify triggers of their intrusive memories, in particular sensory triggers such as visual patterns, colours, shapes, sounds, smells, taste, or touch, and then learn to discriminate the trigger and the context in which they occur in the present (now) from the trauma (then). This involves focusing attention on the differences between the trigger today and its current safe context and the trauma trigger and its past context (Ehlers et al., 2005).

Lommen et al (Lommen et al., 2017) demonstrated experimentally that such perceptual discrimination training reduced the generalisation of fear responses in a differential conditioning experiment. They measured the degree of generalisation of participants’ conditioned responses to graded stimuli (e.g. different shades of grey between CS+(white) and CS− (black)). Participants who had received a perceptual discrimination training were subsequently less avoidant of stimuli similar to the CS− than those who had completed another perceptual task, supporting stimulus discrimination as a possible way to reduce emotional reactions to trauma reminders.

Previous research suggested that stimulus discrimination training may have the potential to reduce intrusive memories and the development of PTSD symptoms after trauma exposure in community samples (Byrne, 2013; Kennedy-Williams et al., 2024). Byrne (2013) and Kennedy-Williams et al. (2024) compared the effects of stimulus discrimination, thought suppression, and a control condition facilitating disengagement (counting) with intrusive memories to traumatic film clips. Participants in the stimulus discrimination condition experienced fewer intrusive memories of the traumatic films and used fewer unhelpful coping strategies in response to unwanted memories than those in the suppression and control group. Kennedy-Williams (Kennedy-Williams et al., 2024) also found fewer reported PTSD symptoms at follow-up for the stimulus discrimination group compared to the other groups.

If stimulus discrimination training effectively reduces the frequency of intrusive memories after trauma exposure and the risk of developing PTSD, such results could inform preventative training programmes for personnel in high risk occupations, such as journalism, emergency medicine and the military. While there is evidence that stimulus discrimination is effective with these populations in the treatment of PTSD (e.g. Ehlers et al., 2014; Ehlers et al., 2023), there is a need to test the efficacy of stimulus discrimination training in highly trauma-exposed samples before exposure to trauma.

In an online experiment, Lorenz (2020) examined the effect of guided training in stimulus discrimination on intrusive memories in student paramedics. In contrast to Byrne (2013) and Kennedy-Williams et al. (2024), this study did not find that the stimulus discrimination training reduced intrusions compared to disengaging from intrusions by counting. However, the author acknowledges that the study did not check whether participants had learned the techniques or were implementing them correctly. The current design therefore includes learning and compliance checks.

At present, there is a gap in the literature on how best to reduce intrusive memories in journalists. The research to date suggests that journalists do experience high levels of intrusive memories (Feinstein & Nicolson, 2005) and tend to respond with suppression-based techniques (Tyson & Wild, 2021). However, the research on whether suppression leads to an increase in memory frequency or can be helpful in decreasing symptoms is mixed (Abramowitz et al., 2001; Mamat & Anderson, 2023; Shipherd & Beck, 2005; Van Schie & Anderson, 2017). Stimulus discrimination, a cognitive technique, has shown promising effects at reducing intrusions (Byrne, 2013; Kennedy-Williams et al., 2024) but has only been tested in community samples.

The current study aimed to address these gaps by comparing training in stimulus discrimination with suppression in a student journalist population in a randomised study. We aimed to determine whether student journalists can be trained to use stimulus discrimination with an online training module, whether training conducted prior to exposure to analogue trauma (trauma films) will result in fewer intrusive memories and fewer PTSD symptoms than training in suppression, and whether there are secondary benefits, such as reductions in depression symptom severity or improvements in reported resilience. The study also explored whether training in stimulus discrimination generalises to unwanted memories of general life stressors.

The primary hypotheses were that participants trained in stimulus discrimination would experience fewer unwanted memories of the trauma films and report less distress associated with them. Secondary hypotheses were that participants trained in stimulus discrimination compared to those trained to suppress unwanted memories would (1) experience less severe PTSD and depression symptoms at 1-week follow-up, (2) would use less suppression and more stimulus discrimination to unwanted memories from general life stressors, and (3) would report more helpful resilience appraisals.

## 2. Methods

### 2.1. Participants

Journalism students aged 18 and over were recruited through lecturers from journalism courses across the UK, Canada, and Australia, who displayed the study advertisement in their classes. Additional recruitment occurred via social media posts from journalism course accounts in these same regions. Participants received £10 for their participation.

Participants were excluded if they scored 10 or higher on the Patient Health Questionnaire (PHQ-9) or 31 or higher on the PTSD Checklist (PCL-5) and were offered support. Since the aim of the study was to prevent PTSD symptoms to a novel stressor, we excluded anyone with preexisting PTSD symptoms above the clinical threshold. This also addressed recommendations from previous research (Weidmann et al., 2009) to exclude those with clinical levels of PTSD and depression symptoms due to the potentially distressing nature of the trauma film paradigm.

**Figure 1.**
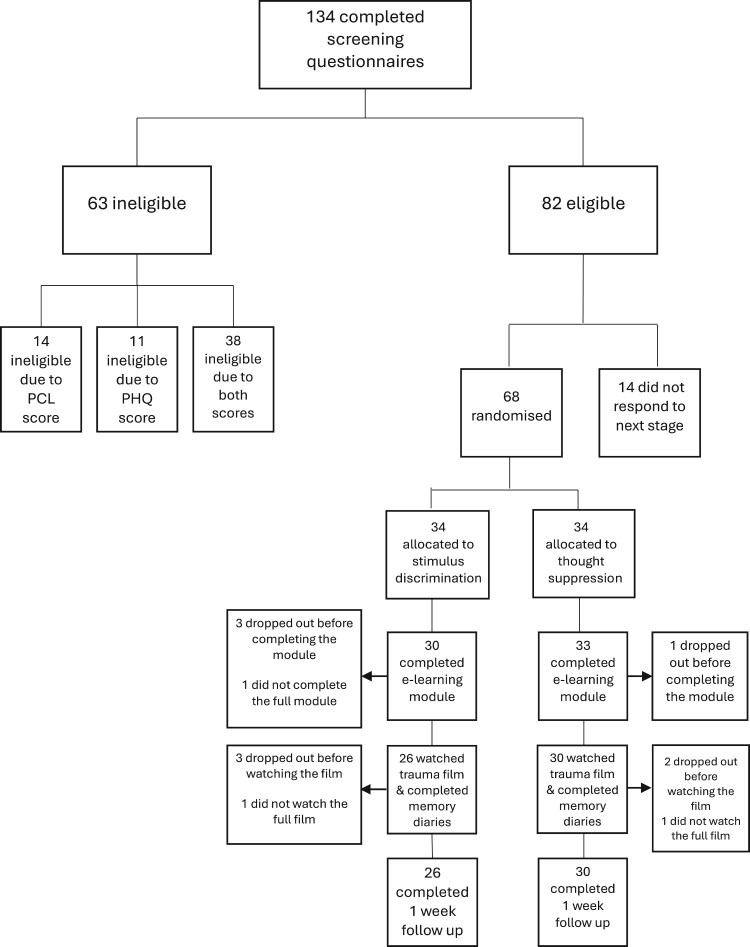
CONSORT diagram.

Figure 1 shows the participant flow through the study. Of 134 people who completed the screener questionnaires, 82 were eligible, and 68 were randomised to one of the training conditions.

The average age of the randomised sample was 23.96 years (SD = 4.35), and 48 were female (71%). There were no differences between the two training conditions on any of the baseline or demographic measures, as shown in Table 1.

**Table 1.** Demographic characteristics and baseline measures.

Measure	Stimulus Discrimination (*N* = 34)		Thought Suppression (*N* = 34)	
	M	SD	M	SD
Age	23.65	4.18	24.26	4.55
Gender: Number of females (%)	24 (70.6)	–	24 (70.6)	–
PCL-5	15.56	10.22	16.47	11.42
PHQ-9	4.97	3.56	5.59	3.24
RIQ Suppression	8.97	2.91	9.68	3.30
CD-RISC	24.03	6.47	26.09	6.23
Distress at films*	71.07	25.96	76.13	18.95

*n = 26 and 30, respectively, there was no significant difference in distress experienced after watching the film between the conditions, *t*(54) = −1.11, *p* = .270.

Note: PCL-5 = PTSD Checklist for DSM-5; PHQ-9 = Patient Health Questionnaire-9; RIQ = Responses to Intrusions Questionnaire; CD-RISC = Connor-Davidson Resilience Scale.

### 2.2. Training conditions

The stimulus discrimination module built on training developed for previous studies (Byrne, 2013; Kennedy-Williams et al., 2024; Lorenz, 2020) with adaptations to make it: (1) fully online and (2) relevant to student journalists. A group of student journalists (*N* = 2) and journalism lecturers (*N* = 3) gave feedback on the modules during the development phase and offered case examples that could be used in the module to demonstrate the techniques.

The stimulus discrimination module begins with a definition of intrusive memories, then guides participants through a series of steps to employ stimulus discrimination, followed by an experiential exercise to practice the technique. Participants view a brief video of a motorbike crash and subsequently hear an audio cue designed to prompt an unwanted memory, providing an opportunity to apply the technique. Participants are prompted to differentiate between NOW (the sensory cue that triggers a memory of the trauma clip in the current safe context) and THEN (the same cue within the original trauma film). Participants are instructed to detect and focus their attention on all of the differences between the current trigger cue and the original trauma context, such as time of day, location, light, colour, and sound (Wild et al., 2018).

The suppression module was developed to teach participants to suppress intrusive memories when they come to mind. It was designed to be as similar in style and duration as the stimulus discrimination module, and to be relevant to student journalists. It begins with a description of intrusive memories using relevant examples and then provides information on PTSD and its symptoms (psychoeducation). Participants are then taught the suppression technique, referred to as ‘controlling memories’, and view the same video clip of a motorbike crash, followed by the identical sensory cue designed to trigger an unwanted memory.

### 2.3. Trauma films

The selection of trauma film clips was based on previous research and pilot testing examining their ability to induce intrusive memories. It was determined that a 7-minute segment from the film ‘The Last King of Scotland’ (Macdonald, 2006), as used in (Marks & Zoellner, 2014) followed by a 15-minute segment from the film ‘Salò or 120 Days of Sodom’ (Pasolini, 1975), as used in (Weidmann et al., 2009), both depicting violence, were most effective at eliciting intrusive memories for 3–7 days following exposure.

### 2.4. Sample size

Power analysis based on Byrne (2013), indicated that, for a large effect size (*f* = 0.40), significance at .05 and power of .90, 34 participants were required per condition for an ANCOVA with two covariates (baseline PTSD symptom severity and gender). Therefore, 68 people were recruited.

### 2.5. Ethics approval

The Central University Research Ethics Committee (CUREC) at the University of Oxford (Medical Sciences Interdivisional Research Ethics Committee) granted ethical approval for the study (R77502/RE001).

See Figure 1 for the CONSORT diagram.

#### 2.2.1. Baseline measures

*Demographics*: Participants were asked to indicate their age, gender, institution where they were studying and year of study.

*PTSD Checklist for DSM 5 (PCL-5)* (Weathers et al., 2013): This 20-item self-report measure of PTSD symptoms as defined by DSM-5 (American Psychiatric Association, 2013) that asks participants to identify a traumatic event and then rate how much they have been bothered by each symptom using a five-point Likert scale from 0 (not at all) to 4 (extremely). The items correspond to the symptom clusters outlined in the DSM-5 (American Psychiatric Association, 2013) (reexperiencing, avoidance, negative cognitions and mood, arousal symptoms). In line with previous research, a clinical cut-off of 31 was used (National Collaborating Centre for Mental Health, 2018). The scale showed good reliability in this sample (Cronbach’s α = .89).

*Patient Health Questionnaire (PHQ-9)* (Kroenke et al., 2001): This 9-item self-report questionnaire is a widely used standard measure of depression symptom severity. Participants rate how much they have been bothered by each depression symptom on a four-point Likert scale from 0 (not at all) to 3 (nearly every day). The PHQ-9 showed good reliability in this sample (Cronbach’s α = .83). Items are summed to give a total score, and a clinical cut-off of 10 was used in the present study, in line with previous research (National Collaborating Centre for Mental Health, 2018).

Participants scoring below clinical cut-offs on the PHQ-9 and PCL-5 completed the following measures.

*The Life Events Checklist (LEC)* (Gray et al., 2004) assessed past trauma exposure by asking participants to indicate whether they had experienced, witnessed or learned about 22 different types of traumatic events. The number of endorsed event types gives a total score.

*Responses to Intrusions Questionnaire – Suppression Subscale (RIQ)* (Murray et al., 2002; Clohessy & Ehlers, 1999): a self-report measure of responses to unwanted memories, participants are asked to rate the frequency in the past week with which they engaged in various strategies for dealing with memories of stressful events from 0 (never) to 3 (always). The RIQ has three subscales: suppression, rumination and numbing responses. This study used the suppression subscale to measure suppression of stressful memories in participants’ day-to-day lives. At follow-up, a further subscale was added to assess the use of stimulus discrimination. The suppression subscale had acceptable reliability (Cronbach’s α = .64); the stimulus discrimination subscale showed good reliability (Cronbach’s α = .80).

*The Connor-Davison Resilience Scale (CD-RISC)* (Connor & Davidson, 2003) asks participants to rate how true statements about their resilience felt to them in the past two weeks on a scale from 0 (not at all true) to 4 (nearly all the time). Items are summed to give a total score. The scale demonstrated acceptable reliability in this sample (Cronbach’s α = .72).

#### 2.2.2. Outcome measures

*Intrusive memory diary (primary outcomes):* Participants were messaged by SMS a link to a diary following the film. The diary asked participants to record the number of intrusive memories experienced that day, a description of the most frequent intrusive memory, the highest level of distress experienced in relation to the memory and the extent to which they were able to apply the technique they learned (on a scale of 0-10).

*Symptom measures (secondary outcomes):* The PCL-5 and PHQ-9 were administered again one week following exposure to the film clip. For the PCL-5, instructions were modified to direct participants to reference the trauma film as the worst event and to complete the questionnaire based on symptoms experienced during the last week.

Resilience and responses to memories of stressful events (secondary outcomes): The RIQ and CD-RISC were given one week following exposure to the film clip. For the RIQ, the instructions remained the same, participants were asked to complete the questionnaire based on how they typically respond to general intrusive memories.

#### 2.2.3. Manipulation checks

*Compliance with training:* As defined in the pre-registered data analysis plan, a manipulation check was carried out for those who viewed the training modules. For the stimulus discrimination training, which contained four exercises to complete, participants were required to answer at least one of these correctly to be included. This resulted in one participant being excluded. The suppression module contained an audio clip and subsequent questions to practice suppression, participants were required to have listened to the audio clip to be included. This did not result in any participants being excluded.

*Subjective Units of Distress Scale (SUDS)* (Wolpe, 1985): To assess the distress induced by the trauma films, participants rated the subjective intensity of their current distress on a scale from 0 (no distress; totally relaxed) to 100 (highest anxiety/distress that you have ever felt). It was given immediately before viewing the film and immediately after.

*Compliance with film viewing:* Questions about the content of the film were given immediately after viewing to measure compliance. Seven participants did not watch the trauma film and were excluded. No participants who watched the trauma film were excluded for failing to answer the questions. Participants were asked to rate how distressing they found the content of the film on a scale from 0 to 100.

*Application of trained method after film:* Within the daily intrusive memory diaries, participants were also asked the extent to which they were able to use their technique for responding to intrusive memories, on a scale from 0 (not at all) to 10 (completely), requiring participants to have used their technique at least 1 out of 10. No participant was excluded on the basis of this check.

#### 2.2.4. Procedure

Participants were directed to a link to read the information sheet and complete the screening questionnaires which took 7 minutes on average to complete (PHQ-9 and PCL-5). Participants were not compensated for time taken on the screener. If eligible, participants were then sent the baseline questionnaires described above. Once these were completed the participant was randomised to the stimulus discrimination or suppression condition. Randomisation by minimisation was stratified on PCL score and gender, using the MS-DOS program titled ‘minim’ (Evans et al., 2021). *N* = 134 completed the screener, *N* = 82 were eligible for the study (see Figure 1 for detail).

Once randomised participants were sent a fifteen-minute online module to complete.

Participants watched the trauma film within 48 hours of completing their respective training. Before viewing the film, participants were reminded of the technique they had learned and were instructed to make the film full screen on their device and to use headphones.

At 8pm each subsequent day, participants were sent a link to an intrusive memory diary to complete, until they no longer reported memories. This follows the recommended procedure (James et al., 2016), that demonstrated the frequency of intrusive memories after exposure to trauma films follows a linear pattern until intrusive trauma memories are no longer experienced. Seven days after watching the trauma film, participants were sent the 1-week follow-up questionnaires, which took on average 10 min to complete.

#### 2.2.5. Analysis

The statistical analysis plan was pre-registered on the Open Science Framework before data collection was completed (osf.io/m3z4c). All analyses were completed in SPSS (version 26.0.0.0).

As outlined in the pre-registration, participants who were non-compliant in terms of (1) did not complete the memory training (stimulus discrimination/suppression) or (2) did not watch the trauma film or (3) did not complete their intrusion diary, were not included in the analysis.

Analysis of covariance was conducted to compare the two conditions on frequency of intrusive memories, subjective distress of intrusive memories, PTSD symptoms, depression symptoms, responses to intrusions and resilience appraisals. Gender and baseline PCL score were the basis for stratification, and thus were included as covariates in all ANCOVAs, as recommended by the European Medicines Agency guidelines (EMA Guideline, 2015). For analyses of resilience, depression and responses to intrusions at 1-week follow-up, baseline scores on each measure respectively were entered as covariates.

Missing data was excluded listwise. One participant had missing CD-RISC data at follow-up and was thus excluded from the analysis for this measure. Significance levels were set at *p* < .05.

## 3. Results

### 3.1. Compliance

As depicted in Figure 1, eight participants in the stimulus discrimination condition were non-compliant (4 did not complete the training, and 4 did not watch the trauma film). Four participants in the suppression condition were non-compliant (1 did not complete the training, and 3 did not watch the trauma film). In both conditions, all those who watched the trauma film went on to complete the intrusive memory diaries. Non-compliant participants were not included in the analysis (*N* = 12 total).

### 3.2. Frequency of intrusive memories of films and associated subjective distress

The ANCOVAs (controlling for the effects of gender and baseline PCL-5 scores) showed a significant effect of training condition on frequency of intrusive memories, F(1,54) = 5.23, *p* = .026, and on the subjective distress associated with them, F(1,54) = 5.64, *p* = .021. Student journalists in the stimulus discrimination condition reported fewer intrusive memories of the film and less distress associated with them than those in the suppression condition.

### 3.3. Symptom scores at 1-week follow-up

The ANCOVAs (controlling for the effects of gender and baseline PCL-5 scores) also showed a significant effect of training condition on PTSD symptom severity scores (PCL-5) in relation to the trauma film at 1-week follow-up, F(1,54) = 7.96, *p* = .007. Participants in the stimulus discrimination condition reported significantly lower PTSD symptom severity scores than those in the suppression condition. There was no significant effect of training condition on depression symptoms (PHQ-9), F(1,54) = 0.63, *p* = .430.

### 3.4. Responses to intrusions of stressful memories in general and resilience at 1-week follow-up

There was no significant effect of training condition on the degree to which participants reported using suppression or stimulus discrimination as a response to stressful memories in general (RIQ): suppression F(1,54) = 0.67, *p* = .574 (when controlling for gender and baseline PCL-5 score); stimulus discrimination F(1,54) = 1.28, *p* = .290 (when controlling for gender and baseline PCL-5 score). Additionally, suppression training showed no significant effect on the use of suppression as a response to stressful memories when controlling for baseline suppression, F(1,54) = 1.89, *p* = .127.

There was no significant effect of training condition on resilience scores (CD-RISC) after controlling for the effects of gender and baseline PCL-5 and resilience scores, F(1,53) = 3.95, *p* = .052. Table 2 shows the means and standard deviations for the primary and secondary outcome measures for each condition.

**Table 2.** Means and standard deviations for outcome measures for participants included in the analysis (*N* = 56).

Measure	Timepoint	Stimulus Discrimination (*N* = 26)		Thought Suppression (*N* = 30)	
		M	SD	M	SD
Frequency of intrusive memories		4.23	3.60	9.17	10.22
Mean distress of intrusive memories		6.31	4.47	9.90	6.57
PTSD symptoms (PCL-5) to trauma film		9.50	6.26	15.73	11.53
Depression (PHQ-9)	Baseline	4.65	3.62	5.70	3.22
	Follow-up	5.54	3.14	6.40	5.67
Resilience (CD-RISC) (stressful events in general)	Baseline	23.73	7.02	26.03	6.16
	Follow-up	23.48	5.82	26.87	5.66
RIQ-suppression subscale (memories of stressful events in general)	Baseline	9.23	2.63	9.33	3.24
	Follow-up	9.54	2.77	10.17	3.39
RIQ-stimulus discrimination subscale (memories of stressful events in general)	Baseline	–	–	–	–
	Follow-up	5.73	2.81	6.03	3.22

Note: PCL-5 = PTSD Checklist for DSM-5; PHQ-9 = Patient Health Questionnaire-9; RIQ = Responses to Intrusions Questionnaire; CD-RISC = Connor-Davidson Resilience Scale.

## 4. Discussion

The aim of this study was to investigate whether trainee journalists could learn to use stimulus discrimination with a one-session online training module and whether stimulus discrimination would be effective at reducing the frequency and distress of intrusive memories following trauma films compared to training in suppression. The results demonstrated that 97% were able to accurately use stimulus discrimination in the online training module and that trainee journalists who received stimulus discrimination training experienced fewer intrusive memories, less associated distress and lower PTSD symptom severity scores following exposure to analogue trauma than those who received suppression training. This finding is consistent with research that has demonstrated the effectiveness of stimulus discrimination in healthy adults (Byrne, 2013; Kennedy-Williams et al., 2024; Lommen et al., 2017).

The effects of stimulus discrimination training appear to be specific to PTSD symptoms. Stimulus discrimination training did not reduce depression symptom scores, which were low at baseline. This finding may be explained by the fact that stimulus discrimination specifically addresses the involuntary triggering of intrusive memories of trauma and does not directly address depressive symptoms. The type of trauma may also be relevant to this result. The analogue trauma used in the study involved witnessing a traumatic scene in which participants had no active role, making it unlikely to elicit negative cognitions, such as self-blame that typically contribute to depressed mood.

No significant difference in trait resilience scores emerged between the two groups at follow-up. This finding is unsurprising given that the study was completed over a two-week period, which likely provided insufficient time for individuals to apply their learning to multiple stressors and for such application to influence resilience-related appraisals. Reviews of resilience research demonstrate that changes in trait resilience typically occur over extended periods, during which individuals have opportunities to implement new strategies; consequently, such research recommends longer follow-up periods for studies investigating resilience (Leys et al., 2020). Additionally, self-report measures of trait resilience may not accurately capture resilient functioning, which might be more precisely assessed through better-than-expected performance across multiple domains following trauma exposure.

There were no group differences in the use of suppression or stimulus discrimination as a response to unwanted memories of life stressors at follow-up. The training in stimulus discrimination or suppression was specific to unwanted memories related to the trauma film and had not generalised to memories of stressful life events by 1-week follow-up. A possible reason is that the training itself did not emphasise the generalisability of the technique to other stressful events. It is also possible that more sessions would be needed to consolidate learning. Research demonstrates that multi-session interventions are most effective at facilitating behaviour change (Chmitorz et al., 2018; Wild et al., 2018), whereas the current intervention consisted of one session.

The current study recruited student journalists for participation. A key rationale for this approach was that learning effective coping strategies early in one’s career increases the odds of staying well, as demonstrated in student paramedic populations (Wild et al., 2016; 2020). The process of recruitment revealed that 39% of the student journalists who registered interest scored well above the clinical threshold on measures of PTSD symptoms, depression symptoms, or both. This prevalence exceeds rates typically observed among qualified journalists, and approaches that found in college students (34.4%) (Cusack et al., 2019). These elevated rates may reflect the confluence of increasing mental health symptoms among young people and potential trauma exposure during journalism training (Dykxhoorn et al., 2024; MacDonald et al., 2017). Further research is needed to examine whether stimulus discrimination training would benefit journalists already established in the field.

Future studies could measure both occupational trauma exposure related to journalism and personal trauma exposure to determine how best to structure journalist-focused interventions. Research of other high-risk professions demonstrates that workers in some fields are most affected by personal trauma (e.g. police), while in others, occupational trauma is most salient (e.g. paramedics) (Wild & Chang, 2022). This distinction has not yet been examined in journalists.

When considering the findings of this experiment alongside Ehlers and Clark’s (Ehlers and Clark (2000) cognitive model of PTSD, the results suggest that stimulus discrimination appeared to reduce the triggering of intrusive memories, and the distress associated with them (Tyson & Wild, 2021). The technique involves learning to focus one’s attention on sensory and contextual differences between the original cues seen during the trauma film (then) and the everyday triggers of memories of the film (now). This effect may counteract two trauma-related processes in PTSD: enhanced perceptual priming (e.g. Sündermann et al., 2013), and the generalisation and reduced extinction of conditioned emotional reactions to perceptually similar stimuli (e.g. Hammell et al., 2020). The reduction in distress when experiencing an intrusive trauma-film memory suggests stimulus discrimination may reduce the ‘here and now’ aspect of intrusions and the associated original emotions (Ehlers et al., 2004; Sündermann et al., 2013).

### 4.1. Limitations

There are a number of limitations to consider. The study design did not include a no-intervention control group; therefore, it is not possible to determine whether stimulus discrimination training is superior to no-training at all. The design choice was made to align with prior study methodologies (Byrne, 2013; Kennedy-Williams et al., 2024) and to test the hypothesis that stimulus discrimination training would be more effective than the most commonly used strategy that journalists report for managing unwanted memories. Future research would benefit from incorporating a no-training condition to differentiate between the effects attributable to stimulus discrimination training and those resulting from natural recovery over time or participants’ existing coping strategies.

The study used analogue trauma as opposed to a naturalistic ‘real-life’ trauma allowing for criticism of the ecological validity of the findings. A trauma film paradigm was selected due to the control it offers over extraneous variables, such as distance from the trauma, and allows comparison to similar studies investigating stimulus discrimination (Byrne, 2013; Kennedy-Williams et al., 2024; Lorenz, 2020). Additionally, reviews of the literature have shown the paradigm to be a valid tool to examine intrusive memories and as a test of proof-of-concept interventions (James et al., 2016).

The study used self-report measures of the frequency and distress of intrusive memories. This requires the participant to report the number of intrusions they have experienced in the previous 24-hour period. Research has demonstrated that participants may not be aware of intrusive trauma-film-related thoughts unless they are specifically probed, which suggests that self-report measures of intrusive memories may underestimate their frequency (Takarangi et al., 2014). However, when currently used methods of recording intrusive memories are compared, research has not shown differences in frequency or distress of reported intrusions (Rattel et al., 2019).

Participants were journalism students who may not have experienced the same level of trauma exposure as practicing journalists. It could be hypothesised that experienced journalists who have relied on alternative strategies for extended periods to manage unwanted memories might find it more challenging to learn to implement stimulus discrimination training. Further research is needed to examine this possibility.

Although participants were screened for clinically significant levels of PTSD and depression symptoms, some participants may have been receiving concurrent treatment, and this was not assessed. Future studies should consider asking participants to report any current mental health treatment to enable inclusion as a covariate in statistical models.

## 5. Conclusion

The current study demonstrates that it is possible to train student journalists to use stimulus discrimination as a response to intrusive memories through an online learning tool. Evidence suggests that this tool, adapted from CT-PTSD, a first-line treatment for PTSD, effectively prevents the chronicity of intrusive memories following analogue trauma exposure in non-clinical samples. Stimulus discrimination training also reduced PTSD symptoms compared suppression training. Targeting the development of intrusive memories using stimulus discrimination may help to prevent the onset and persistence of PTSD (Michael et al., 2005). However, further research is needed to determine whether reducing the frequency and distress associated with unwanted memories in the aftermath of trauma reduces the likelihood of developing PTSD. The stimulus discrimination module developed for this study shows promise as an occupational mental health tool for journalists.

## Data Availability

Data are not made publicly to be in accord with the original ethics proposal approved by the local ethics committee. Anonymized data will be available from the corresponding author on request.
